# Recent advances in understanding the development and function of γδ T cells

**DOI:** 10.12688/f1000research.22161.1

**Published:** 2020-04-29

**Authors:** Alejandra V. Contreras, David L. Wiest

**Affiliations:** 1 Blood Cell Development and Function Program, Fox Chase Cancer Center, R364, 333 Cottman Avenue, Philadelphia, PA, 19111, USA

**Keywords:** γδ T cells, development, function, immune response

## Abstract

γδ T cells are a subset of T cells with attributes of both the innate and adaptive arms of the immune system. These cells have long been an enigmatic and poorly understood component of the immune system and many have viewed them as having limited importance in host defense. This perspective persisted for some time both because of critical gaps in knowledge regarding how the development of γδ T cells is regulated and because of the lack of effective and sophisticated approaches through which the function of γδ T cells can be manipulated. Here, we discuss the recent advances in both of these areas, which have brought the importance of γδ T cells in both productive and pathologic immune function more sharply into focus.

## Introduction and context

The cloning of the T-cell receptor (TCR) γ chain in 1984 led to the discovery of the second major T-cell lineage, γδ T cells ^1– 4^. γδ T cells use the γδ TCR complex to recognize antigen and differ from αβ T cells in numerous ways, including their phenotype, anatomic location, and contribution to host immunity ^5, 6^. Since the discovery of the γδ lineage, immunologists have sought to decipher how it is specified during development and gain a comprehensive understanding of how this enigmatic T-cell subset contributes to host defense. These efforts have led to substantial progress over the past 15 years. Indeed, γδ lineage T cells have been shown to arise from the same progenitor pool as αβ lineage T cells ^7^, and TCR signal strength has been shown to be a key determiner of lineage identity ^8, 9^. Moreover, unlike αβ lineage T cells that exit the thymus as naïve T cells, many γδ lineage T cells have been shown to acquire effector function in the thymus ^10– 14^, and the generation of interleukin-17 (IL-17)-producing γδ cells has been shown to occur primarily during fetal life ^15^. Finally, numerous γδ TCR ligands have been identified, revealing that γδ TCR ligands do not generally require processing or presentation by major histocompatibility complex (MHC) antigens ^16^, and γδ TCR recognition of ligand is more reminiscent of ligand binding by antibody than by the αβ TCR ^17, 18^. However, an exception to this general rule was recently reported, indicating that human γδ T cells are capable of recognizing melanoma-associated antigenic peptides in an MHC-restricted manner ^19^. γδ TCR ligands include a diverse array of unprocessed molecules, such as non-classic MHC class Ib molecules, H2-T10 and H2-T22, lipids presented via CD1 family members, MHC-related protein 1 (MR1), which presents vitamin B derivatives, and annexin A2, a molecule expressed on the cell surface in response to oxidative stress ^20– 23^. Both the capacity of γδ T cells to recognize ligands associated with infection, tissue stress, and transformation and their abundance at epithelial surfaces have direct implications for function, allowing γδ T cells to contribute to host defense through recognizing stress- and tumor-associated molecules expressed by epithelial or tumor cells and promote a rapid stress surveillance response ^6, 24^. Despite the impressive progress noted above, many important and contentious questions remain to be addressed. These include the role of ligand and TCR signaling in controlling γδ T-cell development, the influence of TCR-independent pre-programming on effector fate, and the ultimate contribution of γδ T cells to host defense. Here, we examine recent efforts to address these gaps in understanding.

## Major recent advances

### γδ TCR ligands and their effect on γδ T-cell development and function

One of the primary impediments to a deeper understanding of how γδ T-cell development and function are controlled is the paucity of known γδ TCR ligands, particularly those implicated in development. In recent years, efforts to identify γδ TCR ligands have been increasingly successful, revealing that γδ TCR ligands generally do not require the processing events necessary to generate αβ TCR ligands ^25^. The identity of many of these molecules, such as the non-classic MHC-I molecules H-2T22, as specific ligands has been validated by measures of direct interaction, such as x-ray crystallography or surface plasmon resonance ^26, 27^. However, the legitimacy of another set of putative ligands, the B7-like members of the butyrophilin (BTN) or butyrophilin-like (BTNL) family, was long questioned because of the absence of demonstrable physical interaction with the γδ TCR ^10, 28^. Indeed, murine BTNL family member, Skint-1, is critical for thymic selection of the Vγ5Vδ1 ^+^ subset of dendritic epidermal T cells (DETCs) and their homing to the skin ^29, 30^. Likewise, human family member BTN3A1 is critical for phospho-antigen (p-Ag)-mediated activation of human Vγ9 ^+^Vδ2 ^+^ T cells ^31^. Nevertheless, unequivocal evidence for direct binding of these putative ligands to the γδ TCR was lacking ^25^.

**Figure 1.**
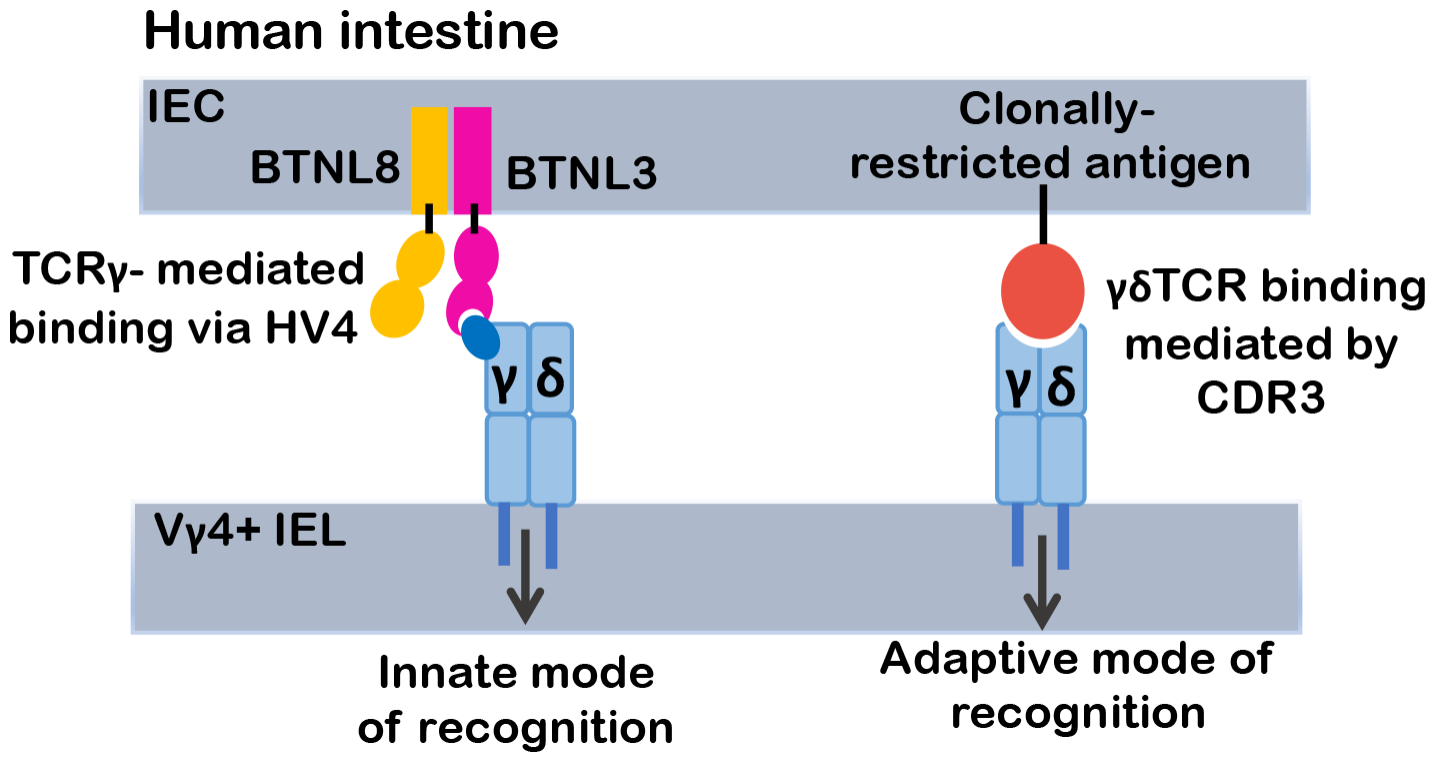
Distinct models of ligand recognition by the γδ T-cell receptor (TCR). Relatively few γδ TCR ligands have been identified. One of the major classes of ligands is the butyrophilin (BTN) or butryophilin-like (BTNL) family. This class of ligands interacts with the γδ TCR in a distinct, CDR3-independent manner that is dependent on framework residues encoded in the germline sequence of TCR-Vγ chains. Consequently, unlike CDR3-mediated ligand recognition that activates only a particular clonotype of γδ T cells, BTN or BTNL ligands are capable of a more innate mode of functioning as they are able to activate all γδ T cells expressing the cognate Vγ chain, irrespective of its TCRγ junctional sequences or its TCRδ subunit. Depicted is the human Vγ4 subunit that binds BTNL3.

Recent efforts have addressed this issue and provided unequivocal evidence supporting the legitimacy of BTNL proteins as γδ TCR ligands and this represents a significant advance in the understanding of how γδ T cells are able to contribute to immunity using both adaptive and innate-like modes of action. Indeed, the Hayday ^32^ and Willcox ^33^ labs determined that the γδ TCR employs two modes of ligand binding: (1) a traditional, clonally restricted mode of binding involving CDR3 sequences that are generated somatically by V(D)J recombination and (2) a CDR3-independent mode of recognition linked to germline-encoded sequences in the V region of the TCRγ chain ( Figure 1). Melandri *et al*. employed a TCR downmodulation assay to demonstrate reactivity of the Vγ7 subunits of murine γδ intraepithelial lymphocytes (IELs) with Btnl1-Btnl6 heterodimers and the reactivity of human Vγ4-containing γδ TCR complexes with BTNL3-BTNL8 heterodimers and to show that this reactivity was dependent on germline-encoded sequences in framework region 3, also known as hypervariable region 4 (HV4) ^32^. Moreover, this mode of ligand recognition, resembling that of superantigen binding by αβ TCR complexes, did not preclude CDR3-mediated clonotypic reactivity with cognate ligand, indicating that individual γδ TCR complexes are capable of both modes of ligand recognition ^32^. Willcox *et al*. reported similar findings and were also able to provide unequivocal evidence for the direct physical interaction between human Vγ4 and BTNL3 ^33^. Importantly, the ability of the human Vγ9Vδ2 γδ TCR to mount BTN3A1-dependent responses to the p-Ag products of the mevalonate pathway was also dependent on the HV4 of Vγ9 ^33^. An additional germline-encoded recognition structure in the Vγ9 subunit also appears to contribute to binding, since a recent report by Rigau *et al*. indicated that Vγ9Vδ2 recognition of p-Ags is also dependent on BTN2A1, which interacts with Vγ9 more through the ABED β-sheet of Vγ9 than through HV4 ^34^. The basis by which p-Ag exposure triggers binding of BTN3A1 to the human Vγ9 ^+^Vδ2 ^+^ TCR has long been a controversial issue; however, recent studies have suggested that the role of BTN3A1 as sensor of p-Ag concentration is mediated through its intracellular B30.2 domain ^35^. In support of this, Yang *et al*. reported crystal structures of the intracellular domain of BTN3A1 protein in complex with the potent microbial p-Ag, (E)-4-hydroxy-3-methyl-but-2-enyl pyrophosphate (HMBPP), revealing that dimerized intracellular domains cooperate in sensing HMBPP and providing insight into the “inside out” triggering of Vγ9Vδ2 T-cell activation ^36^.

The role of ligand in regulating γδ T-cell development remains a contentious issue. The finding that γδ TCR complexes can recognize ligand in both a clonotypic, CDR3-dependent and a Vγ-restricted, HV4-dependent manner at once provides clarification and raises additional questions about the role of ligand in γδ T-cell development. The recognition of Btnl family members, Skint1 and Btnl1-Btnl6, clearly plays an important role in selection of the Vγ5 ^+^ DETC and Vγ7 ^+^ IEL γδ subsets, respectively ^29, 30, 37^. However, the contribution of clonotypic, CDR3-mediated recognition of ligand to γδ T-cell development and the shaping of the repertoire, was more controversial ^14^. Data from TCR transgenic models have provided clear support for the role of CDR3-mediated ligand recognition in the development of murine Vγ4 ^+^ γδ T-cell progenitors reactive with the MHC class 1b ligand, H-2T22 ^9^. Moreover, a recent study indicated that the repertoire of CDR3 sequences (WEGYEL) of polyclonal T-22 reactive γδ T cells that developed in the absence of T22 was markedly altered, suggesting that the CDR3-mediated clonotypic mode of ligand recognition played a positive role in shaping the repertoire of this subset of γδ T cells ^21^. Importantly, whereas this study did not reveal evidence of negative selection induced by CDR3-mediated ligand recognition, previous analysis using transgenic models has suggested that it may occur ^38, 39^. Finally, the γδ T-cell repertoire also appears to continue to be shaped post-thymically since the relatively diverse TCRδ CDR3 repertoire of γδ T cells in human cord blood becomes markedly restricted in adults after viral infection or following reconstitution after hematopoietic stem cell transplantation ^40, 41^. Despite these recent insights into the role of ligand in γδ T-cell development, it remains unclear how extensively CDR3-mediated selection occurs, how CDR3-dependent versus CDR3-independent signals might differ, and the role that these two modes of ligand binding play in determining whether γδ T cells act in an adaptive or more innate-like mode of action in presence of the selective ligand. Addressing the relative contributions of CDR3-dependent and -independent modes of ligand binding to γδ T-cell development and repertoire diversity must await the identification of other CDR3-mediated selection ligands and a determination of whether all Vγ subunits have cognate BTNL family ligands.

### Role of γδ TCR signaling in specification of γδ T-cell effector fate

In addition to commitment to the γδ T-cell lineage, the effector fate of most γδ T cells is determined in the thymus; however, the respective contributions of cellular context and TCR signaling to the specification of effector fate have long been debated. This is a particularly important issue, as γδ T cells can contribute to either host defense or immune-mediated pathology, depending on their effector fate. Indeed, although IL-17–producing γδ T cells are critical for combating infections, aberrant regulation of these cells can contribute to autoimmunity (multiple sclerosis, type 1 diabetes, and psoriasis) and promote tumor progression ^42– 46^. Likewise, interferon gamma (IFNγ)-producing γδ T cells play critical roles in host defense but can also contribute to pathology, such as in cerebral malaria ^47– 49^.

There is clear evidence that TCR signals regulate effector fate. Numerous reports indicate that strong TCR signals, in some cases induced by ligand-engagement of the γδ TCR, promote adoption of the IFNγ-producing effector fate but that the IL-17–producing effector fate depends on weaker γδ TCR signals ^14, 21, 47, 50^. The signals of differing intensity that lead to adoption of these effector fates have been linked to transcription factors (TFs) that are required for effector function. Indeed, IFNγ producers depend on Egr2, Egr3, Id3, and T-bet function whereas IL-17 producers depend on the action of RORγt, SOX13, and c-MAF ^10, 12, 47, 51, 52^. Nevertheless, accumulating evidence suggests that the paradigm of strong and weak TCR signals promoting the IFNγ and IL-17–producing effector fates, respectively, may be too simplistic. Specifically, the Hayday laboratory reported that, unlike the IL-17 producers that develop in response to weak signals in the absence of ligand, some IL-17–producing γδ T cells are dependent on strong TCR signals, as evidenced by the impairment of their development by attenuation of TCR signaling ^11^. Although this report is seemingly at odds with several other studies indicating that the IL-17 fate is incompatible with strong TCR signals ^14, 21, 47, 50^, two recent reports provide a potential explanation for this apparent discrepancy ^46, 52^. The Ciofani laboratory determined that the TF, c-Maf, is required for development of IL-17–producing γδ T cells and determined that c-Maf induction was inversely associated with γδ TCR signal strength, as defined by CD5 induction ^52^; however, c-Maf is also robustly induced by ectopic expression of activated mutants of the signaling molecules, protein kinase C (PKC) and Ras ^52^. Ectopic expression of these activated signaling molecules could not reasonably be described as generating weak signals but might rather be regarded as producing distinct signals compatible with c-Maf induction. Another study, from the Anderson laboratory, suggests that there are two distinct developmental pathways for IL-17–producing γδ T cells and these pathways are distinguished by CD73 expression ^46^. CD73 expression, which is induced by γδ TCR-ligand engagement and strong TCR signals, marks commitment of most γδ precursors to the γδ lineage ^46, 53^; however, some IL-17–producing γδ T cells do not pass through a CD73 ^+^ stage, consistent with their adoption of the IL-17–producing effector fate in response to weaker or distinct TCR signals ^46^. Taken together, these data clearly indicate that γδ TCR signals play an important role in specification of γδ T-cell effector fate but that role remains to be clarified for each effector subset.

**Figure 2.**
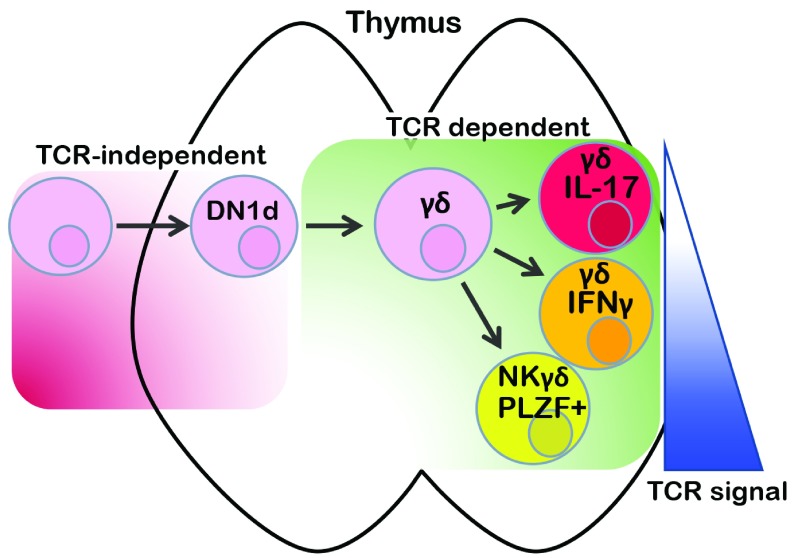
Contribution of cell context and TCR signaling to γδ T-cell effector fate. The relative contribution of TCR signaling and TCR-independent pre-commitment processes to γδ T-cell effector fate has long been debated, particularly regarding the origins of interleukin-17 (IL-17)-producing γδ T cells. Recent lineage tracing studies using a Sox13 reporter revealed that, even before TCR is expressed, cells marked by the Sox13 reporter (stage DN1d) exhibit enriched progenitor activity for IL-17 production. However, that fate potential requires γδ TCR signaling to be manifested and appears to be influenced by the nature of the TCR signal received, with IL-17 production associated with weak TCR signals, the interferon gamma (IFNγ)-producing effector fate being specified by stronger TCR signals, and the strongest TCR signals being required for development of PLZF expressing natural killer (NK) γδ T cells. It should be noted some IL-17–producing subsets can also be induced by strong TCR signals.

There is also evidence in support of cellular context influencing effector fate potential in a TCR-independent manner ^15^. It has been previously proposed that the IL-17–producing effector fate is pre-determined, independent of any influence by TCR signals. Consistent with this perspective, IL-17–producing γδ T cell development is restricted primarily to fetal life ^15^. Moreover, the progenitors of IL-17 producing γδ T cells express the regulatory network of TFs (Sox4, Sox13, Tcf1, and Lef1) that typify IL-17–producing cells at developmental stages that appear to be prior to receipt of TCR signals ^12, 54^. The key experiment necessary to provide insight into the relative contributions of TCR signaling and cellular context to IL-17 effector fate specification was to perform lineage-tracing analysis with an IL-17 fate-defining reporter. Spidale *et al*. employed a Sox13 reporter to identify an early CD4 ^−^8 ^−^ double-negative thymic subset (DN1d) that had not yet expressed γδ TCR, yet exhibited an expression signature linked to the IL-17–producing fate and was enriched in IL-17 progenitor activity ^55^. It should be noted that while the DN1e subpopulation was not marked by the Sox13 reporter, it possessed equivalent progenitor activity for IL-17–producing γδ T cells ^55^. Importantly, development of IL-17–producing γδ T cells from those marked progenitors required TCR signaling, indicating that although the potential to adopt the IL-17–producing effector fate could be established independent of γδ TCR signaling, realization of that potential was dependent on γδ TCR signaling ( Figure 2) ^15, 55^.

### Assessing the functions of γδ T cells

γδ lineage T cells are less abundant in peripheral blood and lymphoid organs than αβ lineage T cells, but γδ T cells are increasingly understood to play a critical role in tissue homeostasis and host defense ^56– 58^. Moreover, localization of γδ T cells at epithelial barriers and their capacity to be rapidly activated make them particularly well suited to be the first line of defense against infections ^57, 59^. γδ T cells can produce large amounts of IFNγ, TNF-α, IL-17, and granzymes and can display pleiotropic immune effector functions ^60^. For instance, the DETC subset of γδ T cells, which produce IFNγ and express high levels of granzymes, can also influence B cells through production of IL-13, regulate stromal cells through production of IGF1, and recruit other leukocytes to the skin by producing chemokines ^6^. γδ T cells have also been shown to be essential to prevent parasitic recurrence in malaria infections ^61^. Surprisingly, γδ T cells producing IL-17 in adipose tissue influence age-dependent regulatory T cell expansion and control core body temperature in response to environmental fluctuations ^62^, whereas those in the meninges of neonatal mice play an essential role in synaptic plasticity and the development of short-term memory ^63^. γδ T cells have emerged as important players in antitumor immunity, showing that many solid tumors and leukemia/lymphoma cells are susceptible to lysis by γδ T cells ^64^. Finally, γδ T cells are able to contribute to immune-mediated pathology, as IL-17–producing γδ T cells have been implicated in the pathogenesis of both psoriasis and multiple sclerosis ^51, 65, 66^. Likewise, IFNγ-producing T cells have been implicated in the pathogenesis of cerebral malaria in mice ^47, 48^ and humans ^49, 67^. Despite this large body of evidence suggesting that γδ T cells contribute extensively to the health of the host, their role in immune responses and host defense remains poorly defined, at least in part because of the lack of effective, consistent strategies with which to acutely eliminate γδ T cells or to selectively modulate their function.

Until recently, efforts to determine whether γδ T cells play an important role in normal or pathologic immune processes have entailed either chronic elimination of γδ T cells using TCRδ-deficiency ( *Tcrd* ^− */*−^) or acute elimination by antibody-mediated depletion. In some cases, these approaches have led to contradictory findings because of their inherent limitations ^68– 70^. TCRδ-deficiency effectively eliminates all γδ T-cell subsets but suffers from the limitation that long-term γδ T-cell depletion enables other cell types to fill the vacated niches and to compensate for γδ T-cell loss, thus leading to the failure to identify key functions. Efforts to acutely deplete γδ T cells suffer from the problem that anti-γδ TCR antibodies do not actually deplete γδ T cells but only downregulate their TCR complexes and make them invisible ^71^. The limitations of both approaches have been circumvented through the development of an effective model of acute depletion of γδ T cells, which involves transgenic expression of diphtheria toxin receptor (DTR) in γδ T cells ^72^. Using this model, the Prinz lab revealed that acute, diphtheria toxin-mediated depletion of IL-17–producing γδ T cells attenuated the development of skin inflammation in a mouse model of psoriasis ^72^. Importantly, the requirement for γδ T cells in pathogenesis in this model was not evident if the mice were allowed to recover for 6 weeks after γδ T-cell depletion; this is because the niches depleted of γδ T cells were repopulated by innate lymphoid cells (ILCs) and IL-17–producing αβ lineage T cells, which were capable of compensating for the loss of γδ T cells and promoting skin inflammation ^72^. The more general use of the DTR transgene to deplete γδ T cells should contribute to advancing our understanding of the role of γδ T cells in normal and pathologic immune responses.

Another limitation that slows progress in understanding γδ T-cell development and function is the paucity of approaches through which γδ T-cell subsets or their functions can be selectively altered. Conditional ablation of key molecular effectors using Cre-recombinase is a standard approach to investigate the function of a particular gene in a cell lineage. For T-cell development, Cre expressed under the control of the proximal Lck promoter-driven Cre ( *pLckCre*) has commonly been used to generate T cell–specific conditional knockout mice; however, Fiala *et al*. ^73^ determined that *pLckCre* does not reliably ablate gene targets in all γδ T-cell subsets at all stages of gestation ^74^. In contrast, *Ptcra*-Cre has been found to reliably ablate gene targets in γδ T cells ^75^. An alternative, which enables temporal control of conditional ablation of target alleles in γδ T cells, is the *Tcrd-CreER* that is expressed in γδ T-cell progenitors and inducible by tamoxifen treatment ^76^. The use of these newly developed tools, and others in progress, to selectively eliminate particular γδ T-cell subsets or alter their effector fates will markedly accelerate progress toward a more comprehensive and unified view of the role of γδ T cells in host health and immunopathology.

### Potential for γδ T cells in human cancer

γδ T cells exhibit many attributes that make them perfectly suited to be anti-cancer effectors ^60^. They are able to infiltrate human tumors and recognize tumor antigens, secrete cytotoxic molecules such as granzyme and perforin, mount rapid cytokine responses without undergoing clonal expansion, and activate adaptive immune responses, all of which make them promising candidates for the development of γδ T cell–based immunotherapies for cancer ^77, 78^. For example, murine γδ T cells have been reported to be effective against cutaneous malignancies ^79^. A recent report revealed that the ability of γδ T cells to resist carcinogenesis in a chemically induced skin cancer model involved regulating the IgE response by B lymphoid cells ^80^. This mode of action may have human relevance since the expression level of the Fc receptor for IgE was linked to outcomes in patients with human squamous cell carcinoma ^80^. Human γδ T cells are able to recognize and kill a broad range of tumor cells, including prostate cancer, melanoma, metastatic renal carcinoma, breast and ovarian cancer, colon carcinoma, hepatocellular carcinoma, lung cancer, and myeloma ^81, 82^. It is likely that particular γδ T-cell subsets exhibit specificity for distinct tumor types. In support of this, the Vδ1 γδ T-cell subset exhibits cytotoxicity against hematopoietic malignancies, melanoma, neuroblastoma, and some other epithelial tumor cells ^81^. The anti-cancer potential of γδ T cells has prompted analysis of their prognostic value in human cancers. Indeed, informatic deconvolution of transcriptomic signatures from a large number (~18,000) of patients with solid tumors revealed that, among immune infiltrates, a γδ T-cell infiltrate is the most favorable prognostic indicator ^83^. More recently, it was reported that the abundance of Vδ1 ^+^ γδ T cells, but not total γδ T cells, was associated with remission in patients with triple-negative breast cancer (TNBC) ^84^. These infiltrating Vδ1 ^+^ cells were enriched for cytotoxic and IFNγ-producing ability and appeared to be functioning in an innate manner, since they were responsive to the NKG2D ligand MICA as well as cytokines IL-12 and IL-18 ^84^.

Despite these encouraging findings that γδ T cells are linked to favorable outcomes in cancer, there are also examples of γδ T cells promoting tumor progression ^68^. In human pancreatic ductal adenocarcinoma (PDAC), in which long-term survival is rare, γδ T cells represent the dominant T-cell population infiltrating the pre-neoplastic pancreas, comprising up to 75% of all T lymphocytes ^85^. γδ T cells appear to promote PDAC progression by inhibiting αβ T-cell activation via expression of immune checkpoint ligand PD-L1 ^85^. γδ T cells have also been shown to promote cancer progression through production of IL-17. IL-17–producing γδ T cells were shown to promote metastasis in a murine breast cancer model by expanding and polarizing neutrophils in the tumor microenvironment ^42^. The activation of IL-17–producing γδ T cells may result from the accumulation of IL-17–polarizing cytokines (IL-1β, IL-6, IL-23, and transforming growth factor-β) in the tumor microenvironment of certain cancers ^24, 42^. Alternatively, the microbiota may also contribute to the capacity of γδ T cells to produce IL-17 and promote tumor progression and metastasis ^86^. In lung, local commensal bacteria have been shown to stimulate the production of IL-1β and IL-23, which induced proliferation and activation of lung-resident Vγ6Vδ1 γδ T cells that produce IL-17 and generate the inflammation associated with lung adenocarcinoma ^87^. These findings highlight the need for a better and more comprehensive understanding of how γδ T cells adopt a specific effector fate, so that anti-tumor function can be favored and the full potential of γδ T cells as anti-tumor effectors can be realized.

## Conclusions

Because of their under-representation relative to αβ lineage T cells in blood and lymphoid organs, γδ T cells were long neglected and thought to play a minor role in host defense. Nevertheless, compelling evidence now suggests that γδ T cells are integral to tissue homeostasis, particularly at epithelial barriers, and are essential to the resistance to infections resulting from barrier breaches. Moreover, they appear to have great potential in cancer both as prognostic indicators and as anti-cancer effectors. Our capacity to exploit the potential of γδ T cells as key contributors to immune responses in general, and in cancer therapy in particular, has lagged behind that of αβ T cells in part because of the lack of appropriate tools to interrogate γδ T-cell function, because of the mistaken impression that γδ T cells are somehow less functionally diverse than αβ lineage cells, and because of a bias that analysis of γδ T cells in mice will not inform their roles in humans. Discoveries described here leave little doubt about the functional diversity of γδ T cells. Moreover, it should be noted that while the particular TCR complexes employed by mouse and human γδ T cells differ substantially, their functional capabilities are quite similar. Indeed, recent evidence strongly supports the existence of human equivalents of the major functional γδ T-cell subsets in mouse (for example, PLZF ^+^ innate γδ T cells, IFNγ producers, and IL-17 producers) ^61, 88, 89^. Accordingly, future efforts to gain a more comprehensive and unified understanding of the role of γδ T cells in human health and disease ultimately will depend on the development of strategies in model organisms to selectively and acutely eliminate particular γδ T-cell subsets to gain insight into their roles. This must be linked to investigation of the molecular processes that control γδ T-cell effector fate so that beneficial and detrimental effector functions can be enhanced and minimized, respectively.

## Abbreviations

BTN, butyrophilin; BTNL, butyrophilin-like; CDR3, complementary determining region 3; DETC, dendritic epidermal T cell; DTR, diphtheria toxin receptor; HMBPP, (E)-4-hydroxy-3-methyl-but-2-enyl pyrophosphate; HV4, hypervariable region 4; IEL, intraepithelial lymphocyte; IFNγ, interferon gamma; IGF1, insulin-like growth factor-1; IL-17, interleukin 17; ILC, innate lymphoid cell; MHC, major histocompatibility complex; NKG2D, natural killer group 2D; p-Ag, phospho-antigen; PDAC, pancreatic ductal adenocarcinoma; PD-L1, programmed death-ligand 1; PKC, protein kinase C; pLckCre, proximal Lck promoter-driven Cre; PLZF, promyelocytic leukemia zinc finger protein; Ptcra-Cre, pre-T-cell antigen receptor alpha-Cre; RORγt, retinoic-acid-receptor-related orphan nuclear receptor gamma; Sox4, SRY-box transcription factor 4; Sox13, SRY-box transcription factor 13; TCR, T-cell receptor; Tcrd ^− */*−^, T-cell receptor delta-deficient; TF, transcription factor; TNBC, triple-negative breast cancer; TNF-α, tumor necrosis factor-alpha.
